# Policy research on role of traditional medicine in emergency health system construction based on the PMC index model: evidence from China

**DOI:** 10.1186/s12906-024-04743-4

**Published:** 2025-01-08

**Authors:** Yujing Zhang, Xia Tian, Zhao Chen, Ziteng Hu, Huizhen Li, Xingyu Zong, An Li, Fuqiang Zhang, Yaxin Chen, Haili Zhang, Lijiao Yan, Ning Liang, Nannan Shi, Yanping Wang

**Affiliations:** 1 Institute of Basic Research in Clinical Medicine, China Academy of Chinese Medical Sciences, Beijing, 100700 China; 2 International Research Center for Medicinal Administration Peking University, Beijing, 100191 China

**Keywords:** Traditional Chinese medicine, PMC index model, Policy health, Quantitative policy evaluation

## Abstract

**Background:**

The integration of traditional Chinese medicine (TCM) into emergency health systems in China serves as a model for global policy development and refining the inclusion of traditional medicine in health emergencies.

**Methods:**

This study investigated 13 public health emergency policies related to TCM released by the Chinese central government from 2003–2023. A PMC(Policy Modeling Consistency) index model was developed combining ROSTCM text mining analysis software. The contents of these policy documents were quantitatively assessed using 10 first- and 40 s-level indicators.

**Results:**

The content analysis results showed that current policies focus on emergency treatment, and that the State Administration of Traditional Chinese Medicine is the issuing authority of the main policies, most of which are issued in the form of a notice. The scoring results for the 13 policies showed that two, five, three, and three policies were rated as excellent, good, qualified, and unqualified, respectively. This indicates that the policy quality related to TCM use in emergency response was normally distributed and generally qualified, although room for further improvement exists; policies should follow the principles of science, reasonableness, and operability, and should be updated in a timely manner with continuous development of the governance period while focusing on the policy content, safeguards, and role measures.

**Conclusion:**

Effective integration of traditional medicine into health emergency policies backed by state institutions is vital. This includes enforcing relevant laws and regulations, establishing multidisciplinary medical teams, and developing integrated medicine strategies that support clinical research and maximize the unique benefits of traditional medicine.

**Supplementary Information:**

The online version contains supplementary material available at 10.1186/s12906-024-04743-4.

## Introduction

We live in a constantly changing world where a sudden public health emergency of international concern (PHEIC) can become a major challenge for global health [1]. The frequency and impact of various infectious diseases and outbreaks have shown an increasing trend with the advancement of science, technology, and globalization. The response to these emergencies have largely relied modern medical treatments, such as vaccines and specific drugs [2]. However, the *WHO Expert Meeting on Evaluation of Traditional Chinese Medicine in the Treatment of COVID-19* [3] issued in 2022 urged the national governments to actively promote an integrated medical model for PHEICs that combines traditional and modern medicine. The World Health Organization (WHO) defines [4] traditional medicine as diverse health practices, approaches, knowledge, and beliefs that incorporate plant-, animal-, and mineral-based medicines, spiritual therapies, manual techniques, and exercises applied singularly or in combination to maintain well-being and treat, diagnose, or prevent illness. Traditional medicines are effective, accessible and affordable [5] providing essential support during emergencies [6] when drugs may be unavailable early in treatment [7]. Integrating traditional medical resources to maximize their advantages remains an unsolved challenge.

During previous PHEICs, various countries endeavored to integrate traditional medicine into emergency management. During the 2003 severe acute respiratory syndrome (SARS) outbreak, traditional Chinese medicine (TCM) was utilized, with affirmed by WHO’s Western Pacific head for blending TCM with modern medicine [8]. In 2019, coronavirus disease 2019 (COVID-19) pandemic, and TCM played a role. China led in issuing TCM treatment policies for COVID-19 and establishing an emergency system for TCM. The State Administration of Traditional Chinese Medicine issued policies for COVID-19 treatment, such as the *Notice on Printing and Distributing the Diagnosis and Treatment Plan for COVID-19 Infection Pneumonia (Trial Third Edition)* [9] and "three drugs and three prescriptions." [10] While achieving over 90% efficacy and gaining international recognition, TCM’s role remains limited in overall emergency management [11]. To strengthen the role of Korean medicine in the prevention and treatment of COVID-19, the Korean Medical Association issued guidelines on January 29, 2020 [12], emphasizing the integration with modern medicine and recommending Korean medicine for confirmed and suspected patients. Despite these efforts, the role of Korean medicine in emergency management remains limited [13]. In India, the Scientific Advisory Committee of the Central Research Committee on Homeopathy under the Ministry of Ayurvedic, Yoga and Naturopathy, Unani, Siddha and Homeopathy adopted a homeopathic approach for COVID-19, designing preventive and treatment programs based on traditional medicine theories, herbal remedies [14]. Moreover, Ayurveda practitioners, through government support, proposed 10 measures to boost immunity via psycho-neuro-immune mechanisms [15]. Although traditional medicine is actively involved in emergency, limitations in personnel roles and legal support weaken its effectiveness [16]. Developing clear policies and frameworks is essential to better integrate traditional medicine into emergency health systems [16].

We aimed to provide a reference for the improvement and refinement of emergency governance policies related to traditional medicine. This study focused on the role and contribution of TCM in the construction of an emergency health system using China as an example. We reviewed policy documents related to the emergency governance of TCM, established a Policy Modeling Consistency(PMC) index model, quantitatively assessed and analyzed the content of the policy documents, and provide suggestions for improvements.

Through this comprehensive and in-depth exploration of emergency governance policies related to TCM, we will provide new perspectives on the integration of traditional medicine into the global health emergency response system. Meanwhile, we expect to generate more attention and discussion to promote the formulation and implementation of relevant policies and further optimize the emergency health system to better respond to future public health events.

## Literature review

### Origins of infectious disease control policies in China

Epidemics have long posed serious threats to human health [17], making infectious disease management a priority throughout China’s history. TCM has contributed methods to prevent and control epidemics, as early as the Song Dynasty, with special laws and organizations created for disease management [18]. However, with the introduction of modern medicine during the late Qing Dynasty and early Republic of China, TCM’s role diminished, and infectious disease treatment increasingly relied on modern medical approaches [19].

After the founding of the People's Republic of China, infectious diseases like smallpox and cholera were prevalent, prompting the government to elevate epidemic prevention. Early policies focused primarily on modern medicine, such as the Law on the Prevention and Treatment of Infectious Diseases established in 1989, which solidified government leadership in managing epidemics and outlined the roles and responsibilities of health authorities. Although TCM was not initially included in this framework, the 2003 SARS outbreak exposed shortcomings in response pathways and funding, leading to policy revisions. By 2004, the law emphasized improved monitoring, early warning, and proposed a legal basis for TCM in disease prevention.

In 2017, the Law of Traditional Chinese Medicine underscored TCM's role in emergencies, though the specifics of TCM's operational mechanisms and emergency resources remained unclear. A 2020 draft revision of the Infectious Disease Prevention and Control Law further advocated for integrating TCM with Western medicine in epidemic control. This evolving framework highlights the need for enhanced TCM operability in emergency management.(Supplementary material 1).

**Fig. 1:**
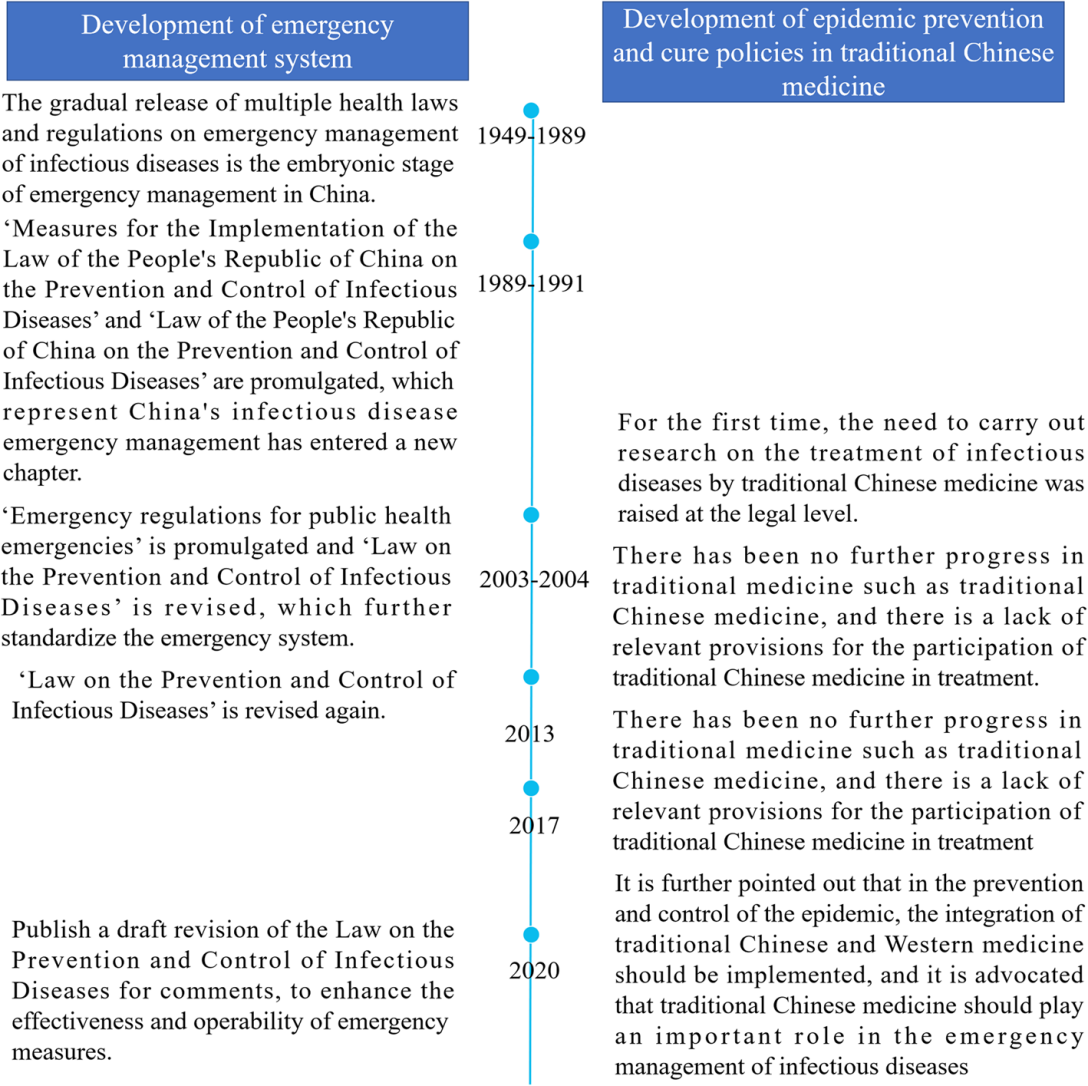
Chronological graph of the emergency management system in China and policy development regarding traditional Chinese medicine governance

With gradual improvements in the national emergency management system, TCM and other types of traditional medicine are urgently needed in distinct roles, as shown in Fig. 1. Fully incorporating the management organization of TCM into decision-making, coordination, and implementation mechanisms is necessary to constantly improve the description of the implementation path and increase the operability of TCM in emergency management.

### Role and implementation difficulties of traditional chinese medicine in emergency management of infectious diseases

During the COVID-19 pandemic, Traditional Chinese Medicine (TCM) demonstrated unique advantages in combating COVID-19. TCM treatment is grounded in the principle of “pattern differentiation and treatment,” which classifies COVID-19 as a “damp-toxin epidemic” or “warm disease” and tailors interventions based on individual symptoms and body constitution. For different symptoms, such as Qi deficiency, damp-heat, or Yin depletion, TCM employs targeted therapies such as clearing heat and detoxifying, tonifying Qi and nourishing Yin, or resolving blood stasis to achieve personalized treatment effects. This individualized approach provides TCM with significant flexibility in addressing infectious diseases with variable symptoms, making it especially suitable for pandemic emergency contexts, as widely validated in clinical practice [20–22]. Li et al. conducted a systematic analysis of 21 studies (including six randomized controlled trials and 15 observational studies), involving 12,981 COVID-19 patients with severe or critical symptoms. The study showed that, compared with conventional supportive care, TCM significantly reduced the risk of mortality, the rate of progression to critical illness, and the use of mechanical ventilation, while also shortening hospital stay, accelerating viral RNA clearance, and speeding symptom resolution [23]. Additionally, no serious adverse events were reported in the TCM group, with safety outcomes comparable to standard care. Similarly, another analysis by Zhu et al., encompassing 59 studies (35 randomized controlled trials and 24 observational studies) and involving 16,580 COVID-19 patients with mild to moderate symptoms, found that TCM significantly reduced the rate of progression to severe cases, shortened symptom and hospital duration, and accelerated viral clearance, all without serious adverse reactions, confirming its safety [24].

The World Health Organization (WHO) explicitly recognized the efficacy and safety of TCM in treating COVID-19 in 2022 and encouraged member states to consider the potential of TCM within their healthcare systems and regulatory frameworks as a treatment option in pandemic response [3]. However, there is still no comprehensive approach to effectively integrate TCM into national healthcare systems. Therefore, reviewing current policies may provide valuable insights for incorporating TCM into future emergency healthcare frameworks.

## Research design

### Sampling

The following databases and websites were searched using the keywords "Traditional Chinese Medicine," "Integrated Traditional Chinese and Western Medicine," "emergency," "COVID-19," "disaster," "disease," "SARS," and "swine flu" to ensure the recall rate: (1) the Chinese government website, National Health and Wellness Commission, and Chinese Center for Disease Control and Prevention (policy documents); (2) CNKI (to collect policy literature); (3) Peking University Laws and Regulations Database (policy texts); and (4) Baidu, as well as other search engines, to conduct final defect detection and filling. The main types of policy texts included in this study were laws and regulations, outlines, plans, opinions, announcements, and other documents. The aforementioned keywords needed to appear multiple times in the policy text, and other document types (such as speeches, meetings, policy interpretations, work reports, review results, project establishment, and opinion responses) were excluded. Finally, according to these consistency requirements, 13 emergency policy documents for TCM were selected as policy research samples, all of which were national-level policies (Table 1).

**Table 1:** Compilation of TCM Prevention and Treatment System for Infectious Diseases

No	File name	Release time	Publishing agency
1	Notice from the State Administration of Traditional Chinese Medicine on Maximizing the Role of Traditional Chinese Medicine in Disease Prevention and Disaster Relief	July 29th, 2003	National Administration of Traditional Chinese Medicine
2	Notice on Maximizing the Role of Traditional Chinese Medicine in Health Emergency Response Work	April 24th, 2009	Ministry of Health P.R.China and National Administration of Traditional Chinese Medicine
3	Notice from the General Office of the Ministry of Health and the Office of the State Administration of Traditional Chinese Medicine on Further Utilizing Traditional Chinese Medicine in the Prevention and Control of H1N1 Influenza (Swine Flu)	January 28th, 2010	Ministry of Health P.R.China and National Administration of Traditional Chinese Medicine
4	Notice from the State Administration of Traditional Chinese Medicine on Issuing the Clinical Research System Construction Plan (Trial) for Traditional Chinese Medicine Prevention and Treatment of Infectious Diseases	November 1st, 2010	National Administration of Traditional Chinese Medicine
5	Notice on Fully Utilizing Traditional Chinese Medicine in Flood Disaster Disease Prevention and Control	July 25th, 2016	National Administration of Traditional Chinese Medicine
6	Notice on Strengthening Information Technology Support for Traditional Chinese Medicine Prevention and Control Work in the COVID-19 Pandemic	February 8th, 2020	National Administration of Traditional Chinese Medicine
7	Notice on Establishing and Improving the Mechanism for Collaboration between Traditional Chinese Medicine and Western Medicine in the Prevention and Control of Infectious Diseases such as COVID-19	February 12th, 2020	National Health Commission of the People's Republic of China and National Administration of Traditional Chinese Medicine
8	Notice on Issuing the Trial Guidelines for Traditional Chinese Medicine Rehabilitation during the Recovery Phase of COVID-19	February 22nd, 2020	National Health Commission of the People's Republic of China and National Administration of Traditional Chinese Medicine
9	Notice from the Office of the State Administration of Traditional Chinese Medicine on Carrying Out Various Tasks of Traditional Chinese Medicine in the Normalized Prevention and Control of COVID-19	May 15th, 2020	National Administration of Traditional Chinese Medicine
10	Notice on Further Strengthening the Prevention and Control of COVID-19 in Traditional Chinese Medical Institutions	June 19th, 2020	National Administration of Traditional Chinese Medicine
11	Notice on Implementing Traditional Chinese Medicine Measures for the Prevention and Control of COVID-19 during the Winter and Spring Seasons	December 14th, 2020	National Administration of Traditional Chinese Medicine
12	Guidelines (Trial) for the Construction and Management of National Traditional Chinese Medicine Emergency Medical Teams	June 15th, 2021	National Administration of Traditional Chinese Medicine
13	Notice on Further Leveraging the Unique Advantages of Traditional Chinese Medicine in the Medical Treatment of COVID-19 Infections	January 2nd, 2023	Joint Prevention and Control Mechanism of the State Council

### PMC-Index model

The Policy Modeling Consistency Index (PMC-Index) is a tool designed to quantitatively assess the internal consistency and content completeness of policy documents. Developed by Mario Arturo Ruiz Estrada under the "Omnia Mobilis" hypothesis, which posits that all elements are interconnected and constantly changing, it moves beyond the traditional "ceteris paribus" assumption [25] by incorporating a wide range of variables in policy evaluation [26]. This ensures that no variable is undervalued, allowing the PMC model to quantify variables within policy texts using binary measurements. By reducing subjective bias, the PMC enhances the scientific rigor and precision of policy analysis [27].

### Data description

#### Text analysis of policy documents

**Fig. 2:**
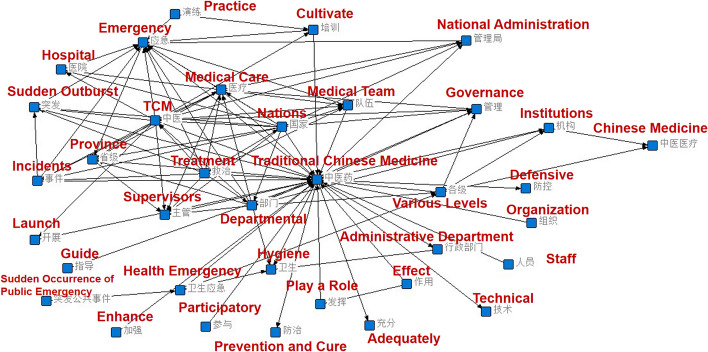
Text mining network diagram of tcm emergency policy

The ROST Content Mining System (ROSTCM) software was used to preliminarily process the 13 policy texts listed in Table 1 and establish a map by extracting high-frequency words (Fig. 2). The policy evaluation indicator system was constructed according to the social network knowledge graph, distribution of high-frequency keywords, relevant policy research results, variable setting method described by Ruiz Estrada, and the indicator settings in policy analysis literature.

#### Selection and setting of variables

Based on the classic framework of the PMC index model and the interpretation of the specific content of the policy documents on the participation of TCM in emergency management, a PMC evaluation index system for the policy documents on the participation of TCM in emergency management was created. The evaluation system was composed of 10 first-level indicators, namely, policy nature, policy time, release agency, function, guarantees measures, perspective, evaluation, receptors, instrument and disclosure, which were further subdivided into 44 s-level indicators, covering as much information and structural elements of the policy documents on the participation of TCM in emergency management as possible, and setting the weight of each second-level variable the same (with = 1, without = 0), to establish a policy evaluation system for public health emergencies. The selection of evaluation indicators and their related explanations and references are shown in Table 2.

**Table 2:** Setting of variables

Main-indicators	Sub-indicators	Source
X1: Policy Nature	X1:1 Forecast	Modified from an article by Estrada
	X1:2 Supervise	
	X1:3 Guide	
	X1:4 Describe	
X2: Policy Time	X2:1 Long-term (> 5 years)	Modified from the article by Lan Yafei, Han Haizhi
	X2:2 Mid-term (3 ~ 5 years)	
	X2:3 Short-term (< 3 years)	
X3: Policy Release Agency	X3:1 the State Council	By authors modifying based on Policy Document information
	X3:2 National Health Commission of the People's Republic of China	
	X3:3 National Administration of Traditional Chinese Medicine	
X4: Policy Function	X4:1 Institution-Building	By authors based on the results from content analysis
	X4:2 Medical Treatment	
	X4:3 Resource Sharing	
	X4:4 Personnel Training	
	X4:5 Expert Organizations	
	X4:6 Clinical Study	
	X4:7 Emergency Plan	
	X4:8 Advantages of TCM	
	X4:9 Informatization	
	X4:10 Responsibility Implementation	
	X4:11 Epidemic Prevention Propaganda	
	X4:12 Hospital Infection Prevention and Control	
X5: Guarantees Measures	X5:1 Personnel Guarantee	By authors based on the results from content analysis
	X5:2 Technical Guarantee	
	X5:3 Organizational Guarantee	
	X5:4 Funding Guarantee	
	X5:5 System	
	X5:6 Law	
	X5:7 R&D Guarantee	
	X5:8 Emergency Supplies	
X6: Policy Perspective	X6:1 Macro-level	Modified from the article by Zhang Yong'an and Qie Hai-tuo
	X6:2 Micro-level	
X7: Policy Evaluation	X7:1 Clear objectives	Modified from the article by Zhang Yong'an and Qie Hai-tuo
	X7:2 Clear authority and responsibility	
	X7:3 Detailed planning	
	X7:4 Scientific program	
X8: Policy Receptors	X8:1 Government Department	Modified from the article by Zhang Yong'an and Qie Hai-tuo
	X8:2 Healthcare institution	
	X8:3 Enterprise	
	X8:4 Social Force	
X9: Policy Instrument	X9:1 Motivational type	Modified from an article by Jinfu Wang and Qingyun Yang
	X9:2 Compulsory type	
	X9:3 Service oriented	
	X9:4 Market oriented	
X10: Policy Disclosure	No sub-indicators	Modified from the article by Zhang Yong'an and Qie Hai-tuo

#### Building a multiple input–output table

In the process of constructing the PMC index model, the multi-input–output table served as an analytical framework for measuring the 10 main variables, each of which consisted of N subvariables (see Table 3 for detailed information).

**Table 3:** Multiple input–output table

Main-Indicators	X1	X2	X3	X4	X5	X6	X7	X8	X9	X10
Sub-Indicators	X1:1	X2:1	X3:1	X4:1	X5:1	X6:1	X7:1	X8:1	X9:1	
	X1:2	X2:2	X3:2	X4:2	X5:2	X6:2	X7:2	X8:2	X9:2	
	X1:3	X2:3	X3:3	X4:3	X5:3		X7:3	X8:3	X9:3	
	X1:4			X4:4	X5:4		X7:4	X8:4	X9:4	
				X4:5	X5:5					
				X4:6	X5:6					
				X4:7	X5:7					
				X4:8	X5:8					
				X4:9						
				X4:10						
				X4:11						
				X4:12						

#### Calculation of PMC index

The formula for calculating the PMC index according to Estrada [26] is as follows:

1 $$\text{X}\sim \text{N}[0, 1]$$

2 $$\text{X}=\{\text{XR}[0\sim 1]\}$$

3 $$\mathrm{Xt}=\left[{\textstyle\sum_{\mathrm j=1}^{\mathrm n}}\frac{\mathrm{Xtj}}{\mathrm T\left(\mathrm{Xtj}\right)}\right].\;.\;\mathrm t=1,2,3,4,5$$

4 $$\text{PMC}=\sum_{\text{t}=1}^{9}\left(\text{Xt}\left[\sum_{\text{j}=1}^{\text{n}}\frac{\text{Xtj}}{\text{T}(\text{Xtj})}\right]\right)$$

In the aforementioned formula, "t" from Formula (3) is the main indicator and "j" from Formula (4) is the sub-indicator.

First, the secondary indicators were graded, calculated, and summarized into the PMC index for policy change, according to the formula shown in Table 4. The maximum score for the 10 first-level indicators was 10 points; therefore, the scores were divided into four levels: 7–10 points, excellent; 6–7 points, good; 5–6 points, acceptable; and < 5 points, poor (Table 5).

**Table 4:** 13 Policy documents’ Scores

No	the scores of main-indicators									
	X1	X2	X3	X4	X5	X6	X7	X8	X9	X10
P1	0.50	0.33	0.33	0.42	0.25	0.50	0.50	0.25	0.25	1.00
P2	0.75	0.33	0.67	0.75	0.88	1.00	1.00	0.75	0.50	1.00
P3	0.75	0.33	0.67	0.50	0.38	0.50	0.75	0.50	0.25	1.00
P4	1.00	0.33	0.33	0.83	0.88	1.00	1.00	0.25	0.75	1.00
P5	0.50	0.33	0.33	0.42	0.25	0.50	0.75	0.25	0.50	1.00
P6	0.50	0.33	0.33	0.50	0.38	0.50	0.50	1.00	0.75	1.00
P7	0.75	0.33	0.67	0.42	0.38	1.00	0.75	0.50	0.25	1.00
P8	0.50	0.33	0.33	0.17	0.13	0.50	0.50	0.50	0.25	1.00
P9	1.00	0.33	0.33	0.67	0.50	0.50	1.00	0.50	0.25	1.00
P10	1.00	0.33	0.33	0.50	0.63	0.50	1.00	0.25	0.25	1.00
P11	0.75	0.33	0.33	0.58	0.63	1.00	1.00	0.50	0.75	1.00
P12	1.00	0.33	0.33	0.50	0.75	1.00	1.00	0.75	0.25	1.00

**Table 5:** Policy Levels

No	the PMC index	The PMC depression index	Evaluation Level	Rank
P1	4.33	5.67	Poor	12
P2	7.63	2.37	Excellent	1
P3	5.63	4.37	Acceptable	10
P4	7.37	2.63	Excellent	2
P5	4.83	5.17	Poor	11
P6	5.79	4.21	Acceptable	8
P7	6.05	3.95	Good	7
P8	4.21	5.79	Poor	13
P9	6.08	3.92	Good	6
P10	5.79	4.21	Acceptable	9
P11	6.87	3.13	Good	4
P12	6.91	3.09	Good	3
P13	6.55	3.45	Good	5

#### Construction of PMC surface

The PMC surface provides a visualization of the advantages and disadvantages of policies. This study included 10 primary variables, of which X10 had no secondary variables, and all policies had a score of 1. Considering the symmetry of the matrix, X10 was removed to form a 3 × 3 square matrix, and Formula (5) was used to identify the PMC surface for the nine policies.

5 $$\text{PMC}-\text{Surface}=\left(\begin{array}{ccc}{\text{X}}_{1}& {\text{X}}_{2}& {\text{X}}_{3}\\ {\text{X}}_{4}& {\text{X}}_{5}& {\text{X}}_{6}\\ {\text{X}}_{7}& {\text{X}}_{8}& {\text{X}}_{9}\end{array}\right)$$

## Result analysis

### Analysis of PMC index

Among the quantitative results for the PMC index for the 13 policies, 3 policies scored < 5 points, which is considered poor, and 10 scored > 5 points. Among these policies, two policies were rated excellent, five were rated good, and three were rated acceptable (Table 5). This indicates that the policy quality of TCM emergency response is normally distributed and generally qualified, although room for improvement remains. Policies should follow the principles of science, rationality, and operability and should be updated along with the flourishing development of governance status.

**Fig. 3:**
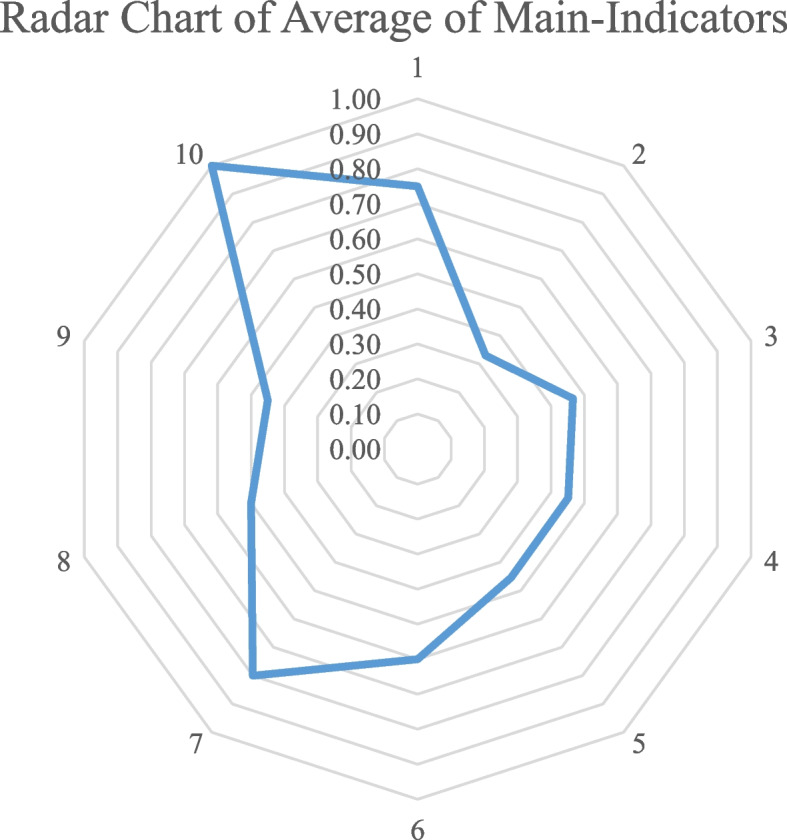
Radar chart of average of main-indicators

The primary indicators were visualized and analyzed using radar charts (Fig. 3). The 10 indicators were ranked from high to low as follows: Policy Disclosure (X10), Policy Evaluation (X7), Policy Nature (X1), Policy Perspective (X6), Policy Receptors (X8), Policy Release Agency (X3), Policy Function (X4), Guarantees Measures (X5), Policy Receptors (X9), and Policy Time (X3). Obviously, the scores of the various policy indicators are uneven and not high, reflecting the overall low level of integration of TCM in emergency response policies. Some weak links remain, providing an objective basis for future policy formulation and revision. The specific discussion is as follows.

#### Analysis of basic policy variables

As shown in Table 6, the Policy Nature (X1) indicator had low prediction and regulatory scores, with low participation observed from common TCM related to pre-warning in emergency policies for public health emergencies. The Policy Perspective (X6) indicator revealed relatively average macro and micro scores, with some policies having both macro indications and micro specific implementation measures. The existing policies are well considered and exhibit strong operability. The Policy Receptors (X8) indicator, revealed that the executing population consisted mainly of government departments and medical institutions with less inclusion of enterprises and the public and insufficient implementation of a joint response by multiple entities. The Policy Receptors (X8) were mainly the National Administration of Traditional Chinese Medicine. Incentives and mandates were the main indicators of action measures (X9).

**Table 6:** Detailed scoring

Main-indicators	Sub-indicators	Scores	Sub-indicators	Scores
X1: policy nature	X1:1 Forecast	0.54	X1:3 Guide	1
	X1:2 Supervise	0.54	X1:4 Describe	1
X2:policy time	X2:1 Long-term (> 5 years)	0.31	X2:3 Short-term (< 3 years)	0.62
	X2:2 Mid-term (3 ~ 5 years)	0.08		
X3:policy release agency	X3:1 the State Council	0.23	X3:3 National Administration of Traditional Chinese Medicine	0.92
	X3:2 National Health Commission of the People's Republic of China	0.15		
X4:policy function	X4:1 Institution-Building	0.85	X4:7 Emergency Plan	0.69
	X4:2 Medical Treatment	1	X4:8 Advantages of TCM	0.46
	X4:3 Resource Sharing	0.46	X4:9 Informatization	0.31
	X4:4 Personnel Training	0.46	X4:10 Responsibility Implementation	0.38
	X4:5 Expert Organizations	0.54	X4:11 Epidemic Prevention Propaganda	0.46
	X4:6 Clinical Study	0.31	X4:12 Hospital Infection Prevention and Control	0.23
X5:guarantees measures	X5:1 Personnel Guarantee	0.85	X5:5 System	0.54
	X5:2 Technical Guarantee	0.85	X5:6 Law	0.15
	X5:3 Organizational Guarantee	0.62	X5:7 R&D Guarantee	0.15
	X5:4 Funding Guarantee	0.23	X5:8 Emergency Supplies	0.69
X6:policy perspective	X6:1 Macro-level	0.62	X6:2 Micro-level	0.77
X7:policy evaluation	X7:1 Clear objectives	1	X7:3 Detailed planning	0.69
	X7:2 Clear authority and responsibility	0.62	X7:4 Scientific program	1
X8: policy receptors	X8:1 Government Department	0.85	X8:3 Enterprise	0.08
	X8:2 Healthcare institution	0.77	X8:4 Social Force	0.31
X9: policy instrument	X9:1 Motivational type	0.77	X9:3 Service oriented	0.31
	X9:2 Compulsory type	0.54	X9:4 Market oriented	0.08
X10: policy disclosure		1		

#### Analysis of policy release time

**Fig. 4:**
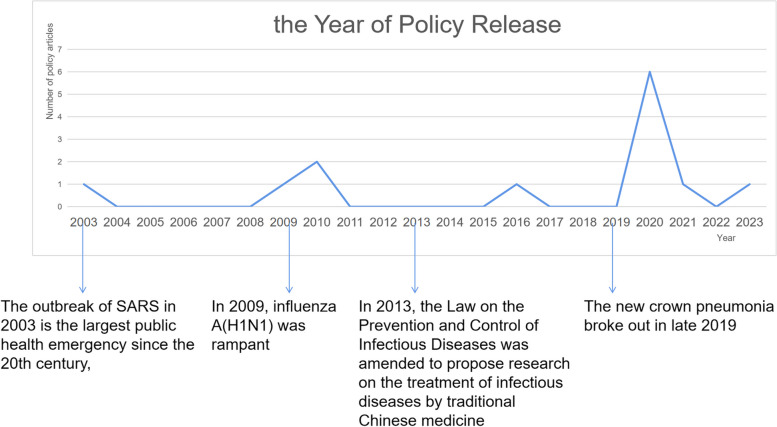
Line chart of policy release time

The Policy Time (X2) indicator had the lowest score because the time of action was one of the three indicators. Owing to the absence of ineffective policies, the policy release time can indicate the effectiveness of the policy (Fig. 4). The first TCM emergency response policy was issued on July 29, 2003 as P1: *Notice of the National Administration of Traditional Chinese Medicine on Giving Full Play to the Role of Traditional Chinese Medicine in Disease Prevention and Disaster Relief*. After the SARS outbreak in 2003, this policy was in the summary and recovery stages. The policy, affirmed the role of TCM in the treatment of SARS, and called for giving full play to the role of TCM in disease prevention and disaster relief in the future. The number of TCM policies for health emergencies increased after the outbreak of influenza A (H1N1) in 2009 and traditional TCM use in health emergencies increased (P2–P4). After the "Infectious Disease Prevention and Control Law" was revised in 2013 to include TCM content, a brief increase in the number of emergency policies for TCM related to hygiene was observed in 2016 (P5). The "Notice on Fully Utilizing the Role of Traditional Chinese Medicine in the Prevention and Control of Diseases in Flood Disasters" did not emphasize disease prevention and control, although focused on emergency policies. At the end of 2019, COVID-19 emerged and spread worldwide, seriously endangering people’s lives and health. The number of policies related to emergency treatment with TCM (P6–P13) reached a peak, and TCM was also recognized as having curative treatment effects.

#### Analysis of basic policy content

The Policy Function (X4) score was average, whereas the scores for institutional construction, medical treatment, and emergency response plans were relatively high. Other scores were relatively low, indicating a lack of resource sharing for the integration of TCM and Western medicine. A shortage of TCM practitioners for epidemic prevention and control was observed, with insufficient attention paid to clinical research on emergency response in TCM. The advantages of TCM related to "syndrome differentiation and treatment" for emergency diseases have not been fully utilized, and TCM has lagged in the trend of informatization. The use of informatization for epidemic prevention and control is insufficient in TCM; strengthening the scientific popularization and promotion of TCM for treating epidemic diseases and preventing and controlling hospital-acquired infections from a macro policy perspective is required. Most scores in the Guarantees Measures (X5) index exceeded 0.5, with a lack of emphasis on guaranteed funding for integrating TCM into emergency management. Relevant laws and regulations and research and development support for TCM in emergency management are insufficient. Although the Policy Evaluation (X7) indicator had the highest score, the scores for the two secondary indicators (clear rights and responsibilities and detailed planning) were average. Therefore, improving the TCM emergency organization system, establishing a TCM emergency management team, clarifying the responsibilities of each unit and department, and planning governance paths for different governance periods in detail is necessary.

P1, *The Notice of the National Administration of Traditional Chinese Medicine on Giving Full Play to the Role of Traditional Chinese Medicine in Disease Prevention and Relief* was the first TCM governance policy. Although rated as unqualified, this policy represents a milestone for TCM regarding inclusion in health emergencies. This incentive macro-policy was issued by the National Administration of Traditional Chinese Medicine to government agencies. In terms of Policy Nature (X1), a description of the early warning effect in TCM was not included, and regulatory measures were not mentioned. Although institutional construction of policy content (X4) indicators occurs, no systematic expert organization is comprehensively responsible for implementing these policies. Since the development of big data and the Internet have lagged behind since 2003, informatization and resource sharing are weak. However, policies have focused on the advantageous role of TCM. For example, TCM plays an important role in the prevention and treatment of orthopedic, respiratory, and infectious diseases. TCM departments at all levels should fully leverage the characteristics and advantages of TCM, actively participate in disaster relief and disease prevention, and improve their emergency mechanisms [28]. Regarding safeguard measures (X5), only personnel protective equipment has been proposed, with emphasis on emergency medical team training. Although no laws and regulations related to the TCM emergency response exist, this policy serves as a normative document related to TCM after the enactment of the Infectious Disease Prevention and Control Law.

Other excellent policy documents identified in this study are P2, *Notice on Giving Full Play to the Role of Traditional Chinese Medicine in Health Emergency Work* and P4 *Notice of the National Administration of Traditional Chinese Medicine on Issuing the Construction Scheme of Clinical Scientific Research System of Traditional Chinese Medicine for the Prevention and Treatment of Infectious Diseases (Trial)*. Both policies scored higher in the Policy Nature (X1) indicator, suggesting that policy could balance the nature of prediction, regulation, advice, and guidance. The Policy Evaluation (X7) indicator received full marks, with clear objectives and detailed scientific plans. These two policy documents describe emergency policies from both macro and micro perspectives, thereby enhancing their operability. The Policy Receptors (X8) in P2 are relatively diverse, whereas P4 only includes one government agency, indicating room for strengthening the collaborative governance among multiple entities. The Policy Function (X4) indicator is relatively comprehensive, comprising 10 aspects, such as the creation of TCM emergency teams and development of clinical research, although lacks a clear understanding of the advantages of TCM and the prevention and control of hospital-acquired infections. Compared with P2, P4 emphasizes the implementation of specific responsibilities among emergency agencies to make the incorporation of TCM into the emergency system orderly. A lack of legal and regulatory guarantees was observed for the safeguard measures (X5) indicator. China vigorously supports the incorporation of TCM into the health emergency system by providing seven guarantees such as personnel, technology, organization, and funding guarantees. However, relevant laws and regulations on the prevention and control of infectious diseases in TCM are urgently required.

### PMC surface analysis

In the previous section, the PMC index results for policies P1, P2, and P4 were discussed in detail. In the following section, we discuss our random selection of P7 and P10 to construct a PMC surface from three dimensions for analysis.

**Fig. 5:**
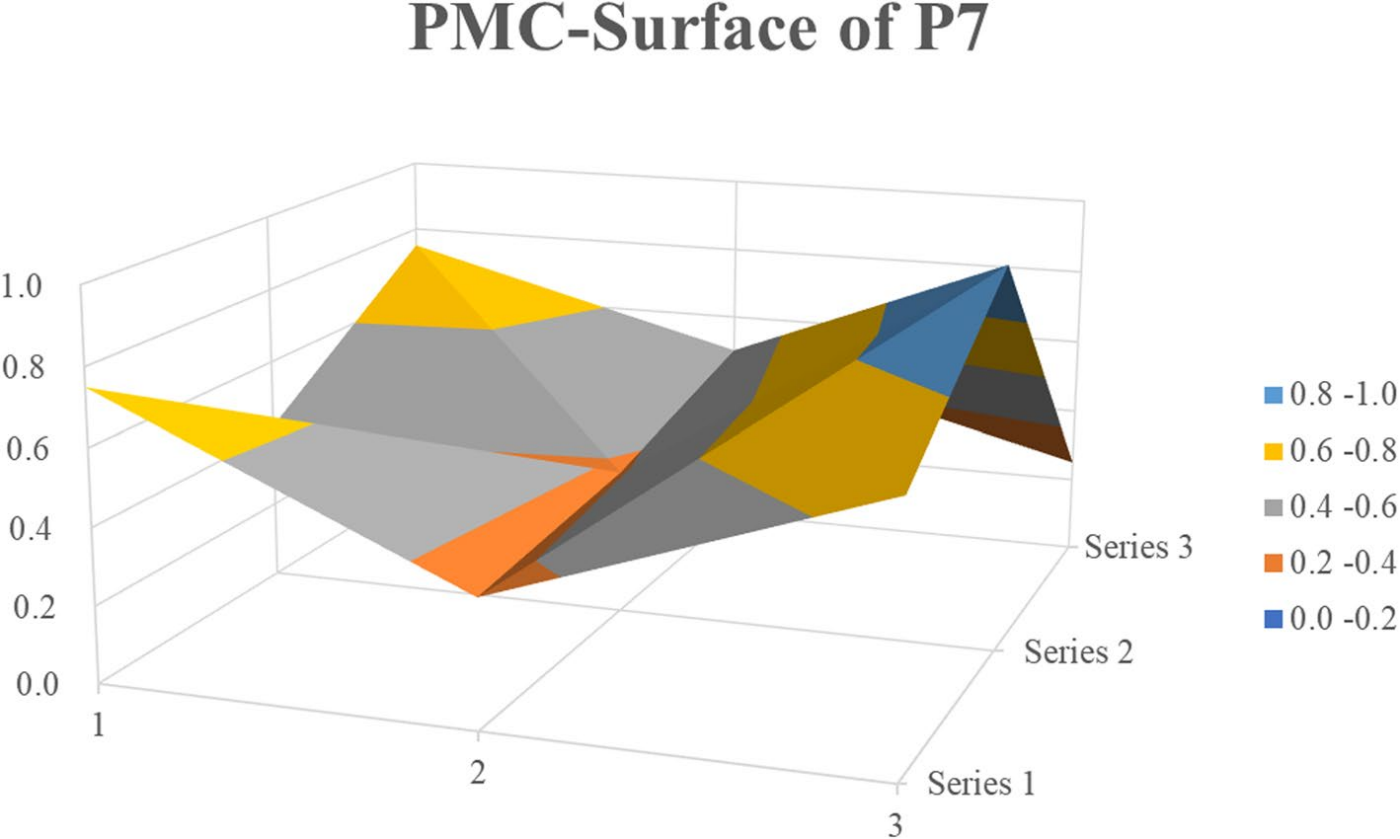
PMC-Surface of P7

P7 is the *Notice on Establishing and Improving the Cooperation Mechanism between Traditional Chinese Medicine and Western Medicine in the Prevention and Treatment of Infectious Diseases such as COVID-19*, issued by the National Health Commission and National Administration of Traditional Chinese Medicine in February 2020 and ranking seventh on the PMC Index. At the beginning of the COVID-19 pandemic, Western infectious disease hospitals were the main source of treatment, and TCM participation was low. Therefore, this normative document was issued to better understand the role of TCM in the prevention and treatment of COVID-19 and other infectious diseases. The surface graph (Fig. 5) shows that the policies at various levels were uneven, with a depression in the middle and two triangular protrusions and depressions around them. This reflected the low scores of the X2, X5, X8, X4, X3, and X9 indicators in the PMC index results. Except for X2, X3, and X8, all the policy scores were low. When analyzing the other indicators, the policy implementation of P1 (incentive method, X9) was limited to the incentive type. The scores for policy content (X4) and safeguard measures (X5) were lower than average because the policy emphasizes institutional construction in emergency situations, expert organization of integrated TCM and Western medicine consultations for medical treatment, and clinical research and resource sharing through the collection of existing cases while neglecting the seven aspects of TCM, such as emergency talent cultivation, responsibility implementation, and informatization, and focuses on personnel, technology, and organizational support while neglecting the five guarantees, such as funds, systems, laws, and regulations. The release of this policy marks the transformation of the anti-epidemic efforts of TCM from a participant to a main force. TCM is deeply involved in the entire diagnosis and treatment process, including early intervention. The integrated diagnosis and treatment of TCM and Western medicine has increased to a national level.

**Fig. 6:**
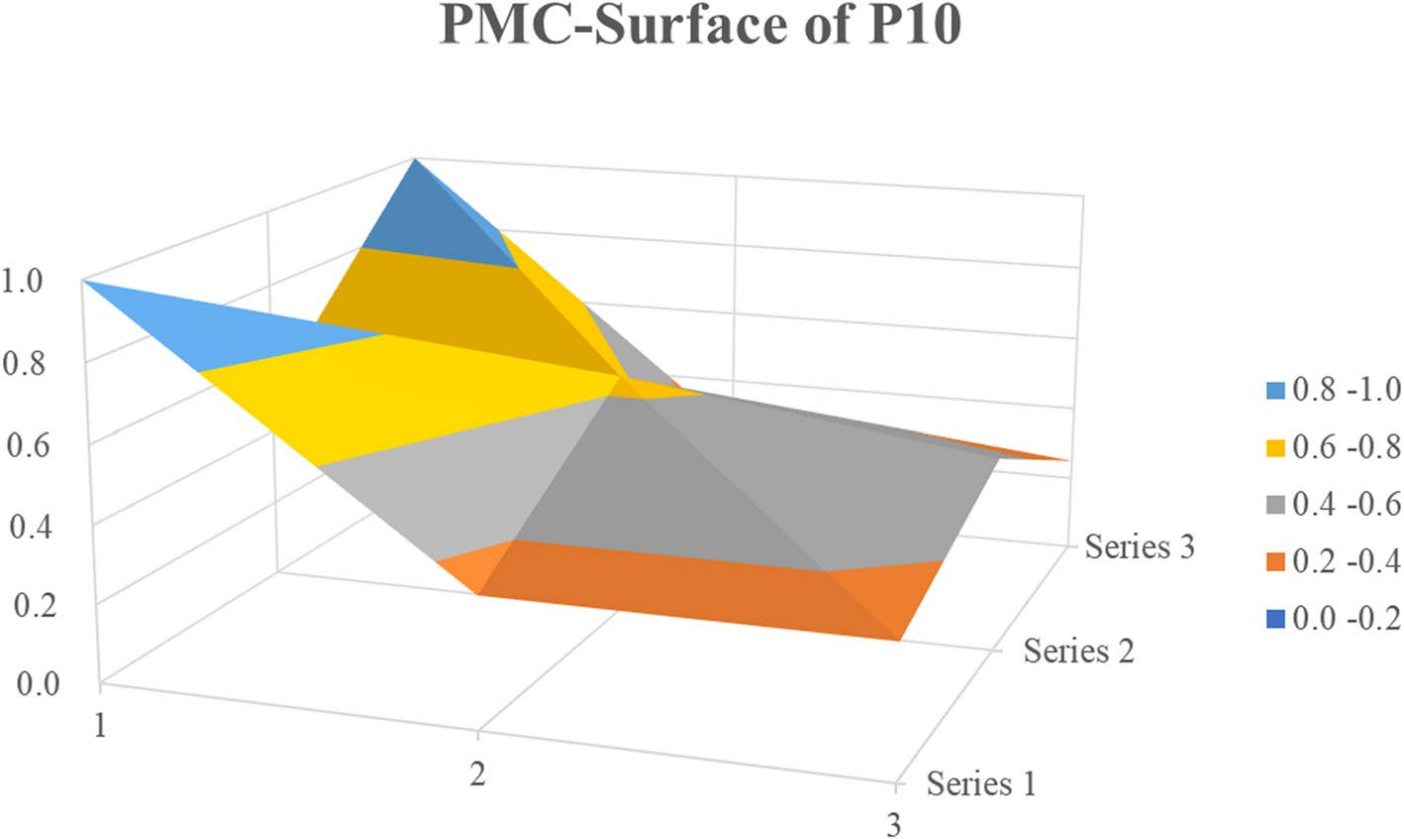
PMC-Surface of P10

P10, the *Notice on Further Strengthening the Prevention and Control of COVID-19 in TCM Medical Institutions* issued in June 2020, ranking ninth on the PMC index. At that time, the COVID-19 situation in China was generally stable, and some provinces and cities had occasional disease clusters. Therefore, this policy was issued to guide TCM medical institutions to further strengthen prevention and control measures for COVID-19, consolidate the hard-won prevention and control achievements, and resolutely prevent the rebound of the epidemic owing to weak local prevention and control. The surface graph (Fig. 6) shows that the surface is high on the left and low on the right. The upper-left corner is raised, and the Policy Nature (X1) indicator had the highest possible score. At the time, the policy considered four aspects: prediction, regulation, suggestion, and description. The Policy Evaluation (X7) score was high. The X2, X3, and X8 indicators had low scores for all policies, with a relatively prominent central area. Compared with P1, the Guarantees Measures (X5) in P10 included institutional guarantees and emergency supplies, aiming to improve the level of care and critical care capacity of TCM hospitals for infectious diseases. Compared with X1, X4, and X7, the left central depression had a lower score for Policy Content (X4). From a micro perspective, policies are released and specific requirements are put forth to further strengthen infection prevention and control measures in TCM medical institutions from six aspects [29]. In addition to the institutional construction and medical treatment of most policies, their indicators include strengthening training and drills for all personnel from a prevention and control perspective; improving talent cultivation for emergency rescue and infectious disease prevention and control capabilities; strengthening the prevention and control of hospital-acquired infection and protection of medical staff; strengthening bottom-line thinking; implementing emergency plans; and strengthening the implementation of role responsibilities related to supervision and inspection efforts.

## Conclusion

Since 2003 SARS outbreak, China's ability to prevent and control infectious diseases has improved. Subsequently, during events like H1N1, Ebola, and COVID-19, the Chinese government issued a series of policies to protect public health and maintain stability. TCM also played a significant role, though its governance impact has been limited by policy inconsistencies. This study analyzed 13 national TCM emergency policies using the PMC index model. The earliest policy in 2003 (*Notice of the National Administration of Traditional Chinese Medicine on Giving Full Play to the Role of Traditional Chinese Medicine in Disease Prevention and Relief*) recognized the governance role of TCM, strengthened its leadership role, and highlighted the unique advantages. The health emergency role of TCM in COVID-19 was described for the first time. Eight (61.5%) governance policies have been issued, mainly by the National Administration of Traditional Chinese Medicine.

The top-ranked keywords in the text analysis were emergency, national, medical, team, department, treatment, hospital, management, prevention and control, organizations at all levels, technology, training, traditional Chinese and Western medicine, administrative departments, diagnosis and treatment, protection, materials, support, and disposal. The emergency governance policies of TCM can be led by the state, with coordinated participation from organizations at all levels and a focus on the treatment and prevention of health emergencies. Establishing TCM emergency teams in a timely manner, integrating TCM and Western medicine, strengthening team training, improving diagnosis and treatment skills, strengthening the development of corresponding research, and coordinating and ensuring emergency supplies is important.

Based on our analysis and summary of the current situation related to emergency policies, the emergency policies for traditional medicine, such as TCM, are summarized as follows.

### Improving construction of laws and regulations

TCM emergency management largely relies on policy documents with limited enforceability. Incorporating TCM and other traditional medicine governance into national laws and regulations, specifying responsibilities, enhancing the prevention and treatment status of TCM from a legal perspective, ensuring the legitimacy and standardization of traditional medicine rescue teams, and improving legal standards for TCM emergency response are urgently required. Finally, strengthening supervision and law enforcement efforts is necessary to ensure the implementation of relevant laws and regulations.

### Highlighting characteristics and advantages of traditional medicine

The unique strengths of TCM, such as personalized treatment, regulating overall balance, and combining prevention and treatment, should be integrated into public health emergency management. Regarding disease prevention in traditional medicine, an auspicious calendar was recorded as early as the Song Dynasty [30]. The decoctions and techniques used in TCM, as well as Indian yoga, should also play a role in emergency management. The early intervention should be performed to avoid transitioning to severe illness, ensuring the health and safety of patients throughout the diagnosis and treatment process..

### Strengthening construction of traditional medicine emergency medical treatment teams

Improving TCM emergency response requires strengthening TCM talent training, conducting emergency drills and enhancing emergency reserves. Information-sharing among health administrative departments, promoting "Internet plus" TCM consultations, and implementing multi-subject collaborative governance are also needed to ensure rapid mobilization and efficient coordination.

### Strengthening integrative medicine models and scientific research innovation

TCM and modern medicine should be fully integrated to enhance treatment effectiveness. Greater emphasis should be placed on the use of TCM and modern medicine combined in response to public health emergencies to facilitate more effective treatment plans and measures than those of TCM or modern medicine alone. Promoting the innovation and application of TCM in the management of public health emergencies is also required, including strengthening research on the prevention and treatment of infectious diseases using TCM, strengthening the research and development of emergency materials and technologies for TCM, and promoting the innovation and development of TCM in emergency management.

According to the example of TCM use in China, the incorporation of traditional medicine(TM) into health emergency policies requires state leadership, implementation of laws and regulations, continuous and comprehensive updates and improvements to existing policy documents, establishment of TM emergency teams, development of emergency plans, grassroots training, continuous expansion of medical teams and strengthening clinical research. Strengthening multi-stakeholder collaboration is crucial to leverage TCM’s full potential within a globalized health system.

### Strengths and limitations

The common methods for policy evaluation models include SWOT analysis, Cost–Benefit Analysis (CBA), and the Balanced Scorecard (BSC). SWOT analysis identifies the strengths, weaknesses, opportunities, and threats associated with the policy [31]. Cost–Benefit Analysis focuses on assessing the economic feasibility and balance of the policy, often by quantifying both the financial benefits and costs involved [32]. The Balanced Scorecard measures policy performance across financial and non-financial dimensions, such as financial outcomes, customer satisfaction, internal processes, and growth potential [33]. In contrast, the PMC index model systematically quantifies policy text across multiple dimensions to objectively evaluate a single policy’s merits and compare its consistency with other policies. By covering a wide array of variables and employing precise binary evaluations, the PMC model offers more objective and reliable results in policy analysis. Given the involvement of multiple stakeholders in the application of traditional medicine within emergency health systems, requiring interdepartmental and multilevel collaboration, using the PMC index model for analysis is reasonable. This study combines qualitative and quantitative methods to analyze the application of Traditional Chinese Medicine (TCM) in existing healthcare policies, providing a foundation and recommendations for countries aiming to integrate TCM into healthcare systems and showcasing a level of innovation in policy evaluation.

However, this study has certain limitations. This paper primarily explores the comprehensiveness and operability of TCM emergency policies from the perspective of policy texts, but policy effectiveness may ideally be evaluated with supplementation of practical outcomes. Evaluating only the policy texts has limitations, especially in analyzing aspects such as policy enforcement strength, changes in the policy environment, public acceptance of TCM, and the interactions between policies and other socio-economic factors, which may still present research gaps.

This study provides insights and references for integrating TCM and other traditional medicine into healthcare systems. Future practice requires further exploration to optimize the evaluation index system, offering more scientific, reasonable, and comprehensive policy recommendations. This would maximize the unique strengths of traditional medicine, support the construction of an integrated medical system, and benefit global public health.

## Supplementary Information

Supplementary Material 1.

## Data Availability

The data for this study are derived from publicly available policy documents on the Chinese government website-- www.gov.cn.

## References

[1] Shuduo ZHOU, Ming XU. On the Global Health Governance in the New World Order and a Suggested Roadmap for China. Modernization of Management. 2022;42(03):114–22. 10.19634/j.cnki.11-1403/c.2022.03.016.

[2] Zhao S, Cao J, Shi Q, et al. A quality evaluation of guidelines on five different viruses causing public health emergencies of international concern. Ann Transl Med. 2020;8(7):500–500. 10.21037/atm.2020.03.130. 32395544 10.21037/atm.2020.03.130 PMC7210117

[3] Organization WH. WHO Expert Meeting on Evaluation of Traditional Chinese Medicine in the Treatment of COVID-19. March 31 2022. Available from: https://cdn.who.int/media/docs/default-source/traditional-medicine/meeting-report---who-expert-meeting-on-evaluation-of-tcm-in-the-treatment-of-covid-192f7d2ba2-cfb8-4b00-90e3-441740cdbacb.pdf?sfvrsn=a77161d7_1&download=true

[4] Organization WH. Programme on Traditional Medicine. WHO traditional medicine strategy 2002–2005. 2002;1, 7. Available from: https://apps.who.int/iris/handle/10665/67163

[5] LI Yi-long, LIU Yi, BIAN Yue-feng, et al. Analysis on the global development of traditional medicine. China J Tradit Chin Med Pharm. 2020;35(07):3578–81.

[6] Yuan ZI, Cheng-xi WANG, Xin-bin XIA, et al. Discussion on the Construction of Prevention and Control System for Public Health Emergencies by Chinese Medicine. Guiding Journal of Traditional Chinese Medicine and Pharmacy. 2020;26(12):213–6. 10.13862/j.cnki.cn43-1446/r.2020.12.058.

[7] Xin DENG, Li-jing LIU, Ya-xu PANG, et al. Research and reflection on the integration of TCM into the emergency system of public health emergencies. Chin J Public Health Manage. 2022;38(05):592–5. 10.19568/j.cnki.23-1318.2022.05.0005.

[8] The World Health Organization fully affirms for the first time the combination of Chinese and Western medicine in the treatment of SARS. October 14 2003. Available from: http://lianghui.china.com.cn/chinese/zhuanti/feiyan/421334.htm

[9] Chinese Government Website. Circular on the Issuance of the Diagnostic and Treatment Program for Pneumonia Infected by New Coronaviruses (Trial Version 3). January 22 2020. Available from: https://www.gov.cn/zhengce/zhengceku/2020-01/23/content_5471832.htm

[10] Novel Coronavirus Pneumonia Prevention and Control Group, Zhongnan Hospital, Wuhan University, Wuhan, China.A clinical diagnosis & treatment rapid advice guideline for integrating Chinese and Western medicine of COVID-19.*Chinese Research Hospitals*.2020,7(02):51–64.10.19450/j.cnki.jcrh.2020.02.013.

[11] The State Council lnformation Office of the People's Republic of China. International Community Positively Evaluates Chinese Medicine Against Epidemic. March 24 2020. Available from:http://www.scio.gov.cn/gjgz_0/202209/t20220921_436058.html

[12] Korean Medical Association. Active use of Oriental medicine for prevention and treatment of COVID-19. January 29 2020.Available from:https://nikom.or.kr/koms/html.do?menu_idx=2

[13] Korean Medical Association. Acceptance of the Korean Medical Association to overcome COVID-19 infections and active use of Oriental medicine in the prevention and treatment of COVID-19. January 29 2020. Available from:https://nikom.or.kr/koms/html.do?menu_idx=2

[14] PIB Delhi. Advisory for Corona virus January 29 2020. Available from:https://pib.gov.in/PressReleseDetail.aspx?PRID=1600895.

[15] Rajkumar RP. Ayurveda and COVID-19: Where psychoneuroimmunology and the meaning response meet. Brain Behav Immun. 2020;87:8–9. 10.1016/j.bbi.2020.04.056. 32334064 10.1016/j.bbi.2020.04.056 PMC7175849

[16] Yufeng Z, Huaxin P, Lanting L, et al. Risk assessment and analysis of Traditional Chinese Medicine intervention in coronavirus disease. J Tradit Chin Med. 2022;42(3):472–8. 10.19852/j.cnki.jtcm.20220408.001. 35610019 10.19852/j.cnki.jtcm.20220408.001 PMC9924655

[17] XIONG Yi-liang, SUN Xin, XUE Han-li, et al. Review on the study of ancient Chinese epidemic literature. China J Tradit Chin Med Pharm. 2022;37(08):4259–62.

[18] Chengwen LI, Min LU, Zhaolin LU. The Influence of the Traditional Chinese Medicine Policy of the Northern Song Government on the Development of Traditional Chinese Medicine. Journal of Beijing University of Traditional Chinese Medicine. 2005;06:26–8.

[19] Xianzhong HAO. A Study of the Controversy over the Abolition and Existence of Traditional Chinese Medicine in Modern Times. Shang Hai: East China Normal University; 2005.

[20] Huang K, Zhang P, Zhang Z, Youn JY, Wang C, Zhang H, Cai H. Traditional Chinese Medicine (TCM) in the treatment of COVID-19 and other viral infections: Efficacies and mechanisms. Pharmacol Ther. 2021;225: 107843. 10.1016/j.pharmthera.2021.107843. 33811957 10.1016/j.pharmthera.2021.107843 PMC8011334

[21] Ren L, Xu Y, Ning L, Pan X, Li Y, Zhao Q, Pang B, Huang J, Deng K, Zhang Y. TCM2COVID: A resource of anti-COVID-19 traditional Chinese medicine with effects and mechanisms. iMeta. 2022;1(4):e42. 10.1002/imt2.42. Advance online publication. 36245702 10.1002/imt2.42 PMC9537919

[22] Zhao Z, Li Y, Zhou L, Zhou X, Xie B, Zhang W, Sun J. Prevention and treatment of COVID-19 using Traditional Chinese Medicine: A review. Phytomedicine : international journal of phytotherapy and phytopharmacology. 2021;85: 153308. 10.1016/j.phymed.2020.153308. 32843234 10.1016/j.phymed.2020.153308 PMC7439087

[23] Li M, Zhu H, Liu Y, Lu Y, Sun M, Zhang Y, Shi J, Shi N, Li L, Yang K, Sun X, Liu J, Ge L, Huang L. Role of Traditional Chinese Medicine in Treating Severe or Critical COVID-19: A Systematic Review of Randomized Controlled Trials and Observational Studies. Front Pharmacol. 2022;13: 926189. 10.3389/fphar.2022.926189. 35910363 10.3389/fphar.2022.926189 PMC9336221

[24] Zhu H, Li M, Tian C, Lai H, Zhang Y, Shi J, Shi N, Zhao H, Yang K, Shang H, Sun X, Liu J, Ge L, Huang L. Efficacy and safety of chinese herbal medicine for treating mild or moderate COVID-19: A systematic review and meta-analysis of randomized controlled trials and observational studies. Front Pharmacol. 2022;13: 988237. 10.3389/fphar.2022.988237. 36160412 10.3389/fphar.2022.988237 PMC9504662

[25] Ruiz Estrada, M. A. , Yap, S. F. , & Nagaraj, S. . (2010). Beyond the ceteris paribus assumption: modeling demand and supply assuming omnia mobilis. Social Science Electronic Publishing.

[26] Estrada MA, Ruiz,. Policy modeling: Definition, classification and evaluation. Journal of Policy Modeling. 2011;33(4):523–36.

[27] 张永安,耿喆.我国区域科技创新政策的量化评价——基于PMC指数模型[J].科技管理研究,2015,35(14):26–31.

[28] Chinese Government Website.The Chinese Medicine Bureau asked to actively play the role of Chinese medicine in disaster relief and disease prevention. February 13 2008.Available from:https://www.gov.cn/govweb/gzdt/2008-02/13/content_889014.htm

[29] Bei HUANG.Circular on Further Consolidation of Achievements in Improving the Prevention, Control and Treatment Capabilities of Medical Institutions for New Crown Pneumonia Issued and Published.Journal of Traditional Chinese Medicine Management. 2020,28(08):5.10.16690/j.cnki.1007-9203.2020.08.003.

[30] Zhe LI, Jinshan LV. On the Desirability of the Main Medical and Health Policies of the Song Dynasty. Chinese Journal of Information on Traditional Chinese Medicine. 2010;17(S1):5–7.

[31] Gurl, E. (2017). SWOT analysis: A theoretical review.

[32] Jiang W, Marggraf R. The origin of cost–benefit analysis: a comparative view of France and the United States. Cost Eff Resour Alloc. 2021;19:74. 10.1186/s12962-021-00330-3. 34794465 10.1186/s12962-021-00330-3 PMC8600932

[33] Kumar S, Lim WM, Sureka R, Jabbour CJC, Bamel U. Balanced scorecard: trends, developments, and future directions. RMS. 2024;18(8):2397–439.

