# Supplementary material for: Policy research on role of traditional medicine in emergency health system construction based on the PMC index model: evidence from China
Source: BMC Complement Med Ther. 2025 Jan 8;25:4. doi: 10.1186/s12906-024-04743-4 (PMC11708103; doi:10.1186/s12906-024-04743-4)
Supplement: Supplementary file 1 — Supplementary Material 1. [file 12906_2024_4743_MOESM1_ESM.docx]

**Supplementary material 1**

| **Time/Event** | **Policies and Measures** | **Description** |
| --- | --- | --- |
| **the founding of the People's Republic of China** | Issued various health laws, such as the *Instruction on Launching the Autumn Vaccination Campaign*, *Measures for the Management of Infectious Diseases* and *Regulations of the China on the Management of Acute Infectious Diseases*. | Focuses mainly on modern medical tools and does not include preventive and control measures in Chinese medicine |
| **1989** | Enactment of the *Law of the China on the Prevention and Treatment of Infectious Diseases* | Government takes legal ownership of infectious disease prevention and emergency management, standardising responsibilities between government and frontline staff |
| **1991** | Ministry of Health issued the *Measures for the Implementation of the Law of the China on the Prevention and Control of Infectious Diseases* | The legal framework for the prevention and control of infectious diseases has been systematised, covering prevention, reporting, disclosure, control and surveillance |
| **2003 SARS Outbreak** | the *Infectious Disease Prevention and Control Law* exposed shortcomings, including deficiencies in monitoring, early warning and communication of information, and the State Council issued the *Emergency Regulations for Public Health Emergencies* | Emergency response mechanisms have been improved and the development and improvement of public health emergency policies have been strengthened |
| **2004** | Revised the *Law on the Prevention and Control of Infectious Diseases* by the Standing Committee of the National People’s Congress to enhance emergency response, sectoral responsibilities, and monitoring and early warning mechanisms | Emphasized early warning systems, control measures, and supported TCM research for the first time |
| **2013** | Further revision of the *Law on the Prevention and Control of Infectious Diseases* | Limited further progress in Chinese medicine in emergency response and lack of clear provisions for Chinese medicine involvement |
| **2017** | Enactment of the *Law of the People's Republic of China on Traditional Chinese Medicine* | Emphasized TCM’s role in public health emergencies, called for TCM supply reserves, but lacked specific operational mechanisms |
| **2020** | National Health Commission’s draft revision of Infectious Disease Law | Strengthening the operationalisation of the law to promote the effectiveness of emergency measures; promoting the full use of Chinese medicine in prevention and control |
